# Crossed wires: diatom phosphate sensing mechanisms coordinate nitrogen metabolism

**DOI:** 10.1080/15592324.2024.2404352

**Published:** 2024-10-02

**Authors:** Yasmin Meeda, Ellen Harrison, Adam Monier, Glen Wheeler, Katherine E. Helliwell

**Affiliations:** a Biosciences, Faculty of Health and Life Sciences, University of Exeter, Exeter, UK; b Marine Biological Association, Citadel Hill, Plymouth, UK; c Living Systems Institute, University of Exeter, Exeter, UK

**Keywords:** Nutrient signaling, diatoms, nitrogen, phosphorus, Ca^2+^ signaling

## Abstract

Phytoplankton can encounter dynamic changes in their environment including fluctuating nutrient supply, and therefore require survival mechanisms to compete for such growth-limiting resources. Diatoms, single-celled eukaryotic microalgae, are typically first responders when crucial macronutrients phosphorus (P) and nitrogen (N) enter the marine environment and therefore must have tightly regulated nutrient sensory systems. While nutrient starvation responses have been described, comparatively little is known about diatom nutrient sensing mechanisms. We previously identified that the model diatoms *Phaeodactylum tricornutum* and *Thalassiosira pseudonana* use calcium (Ca^2+^) ions as a rapid intracellular signaling response following phosphate resupply. This response is evident only in phosphate deplete conditions, suggesting that it is coordinated in P-starved cells. Rapid increases in N uptake and assimilation pathways observed following phosphate resupply, indicate tight interplay between P and N signaling. To regulate such downstream changes, Ca^2+^ ions must bind to Ca^2+^ sensors following phosphate induced Ca^2+^ signals, yet this molecular machinery is unknown. Here, we describe our findings in relation to known diatom P starvation signaling mechanisms and discuss their implications in the context of environmental macronutrient metadata and in light of recent developments in the field. We also consider the importance of studying phytoplankton nutrient signaling systems in the face of future ocean conditions.

Diatoms are unicellular photosynthetic eukaryotes found in marine and freshwater systems. Known for their ability to form spatially extensive blooms in the ocean, diatoms are important drivers of ocean carbon cycling, contributing around 40% of marine primary productivity.^1^ During phytoplankton blooms, diatoms are often early responders to influxes of important nutrients, including nitrogen (N) and phosphorus (P). The concentrations of such macronutrients in the ocean can fluctuate substantially over different temporal scales (Figure 1a,b). Interannual changes in temperate coastal ecosystems typically show increased P and N availability in winter that gradually decline during spring and summer months as diatom populations increase (Figure 1c). Additionally, more rapid nutrient pulses (on timescales from days to weeks) driven by episodic additions such as riverine inputs following heavy rainfall are also apparent, particularly during summer.^2^ In order to compete in such dynamic nutrient environments, diatoms must have mechanisms enabling them to both survive prolonged periods of nutrient limitation, but also detect and rapidly alter their physiology when nutrients become replenished.

**Figure 1.**
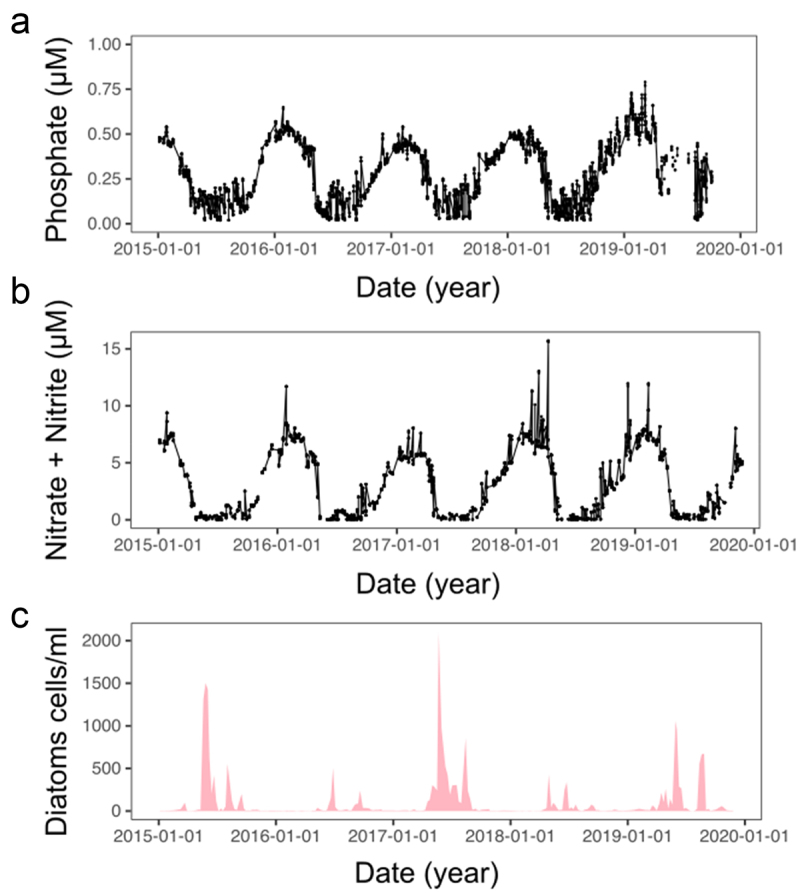
Inorganic phosphate and nitrogen levels are tightly coupled in coastal ecosystems and are major drivers of diatom abundance.

Phosphate (a),nitrate and nitrite (b) concentrations (µM) and total diatom abundance (cells/ml) (c) recorded weekly at coastal L4 station from the Western Channel Observatory dataset between 2015 and 2020. Data available via westernchannelobservatory.org.uk.

## Diatom survival during P limitation

**Figure 2.**
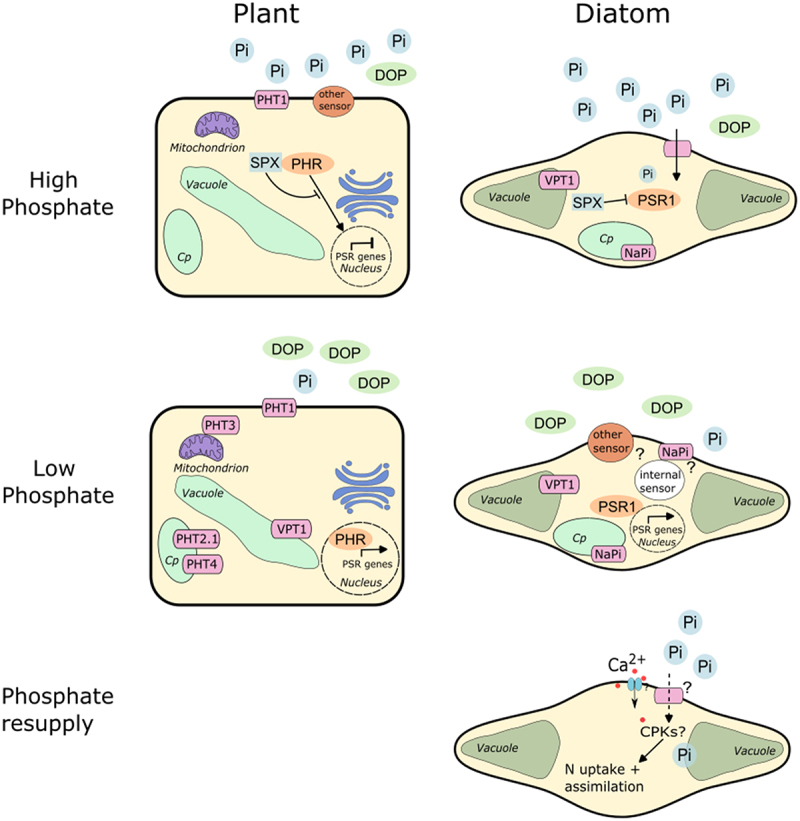
Comparison of phosphate sensing and signalling mechanisms in plants and diatoms.

Examples of how diatoms cope with limited P availability include remodeling phospholipids, which are substituted for non-P containing lipids,^3^ alkaline phosphatases that can scavenge P from organic P containing compounds are induced. Additionally, there is upregulation of phosphate transporters,^4–6^ enabling rapid depletion of extracellular phosphate within minutes following phosphate resupply.^7^ The transcription factor Phosphate Starvation Response 1 (PSR1) in the model pennate diatom *Phaeodactylum tricornutum* has been identified to coordinate P starvation responses and shares homology and functionality with PSR1 of green algae and plants (known as PHR1)^8^ (Figure 2). Like plants, *P. tricornutum* also encodes an SPX (SYG1/Pho81/XPR1) protein that is thought to negatively regulate P starvation responses through interactions with PSR1.^13,14^ Whether this protein senses intracellular inorganic P stores (such as inositol polyphosphates) in a similar manner to other eukaryotes^18^ remains an exciting area for future diatom research.

Despite being united by their ability to photosynthesize, land plants (Archaeplastida) and diatoms (stramenopiles) have vastly different evolutionary histories and ecological lifestyles,^9^ and therefore differences in P starvation signaling and sensing mechanisms are not unexpected. Yet, both taxa do share an apparently conserved master regulator of phosphate starvation responses, Phosphate Starvation Response 1 (so-called ‘PSR1’ (diatoms) or ‘PHR’ (plants)), which belong to the GARP (GOLDEN2/ARR-B/Psr1) family of transcription factors.^10–12^ SPX (SYG1/Pho81/XPR1) proteins also act as negative regulators of PHR/PSR1 in plants and diatoms.^13,14^ Additionally, a homologue of the plant vacuolar phosphate transporter VPT1^15^ has also been identified in *P. tricornutum*.^6^ Several (likely Na^+^-dependent) phosphate transporters have been localized both to the plasma membrane and chloroplast in *P. tricornutum*.^7^ Pi: inorganic phosphate; DOP: dissolved organic phosphorus; PSR: phosphate starvation response; CPKs: calcium (Ca^2+^) dependent protein kinases; PHT: phosphate transporter; Cp: chloroplast. Phosphate sensing and signaling mechanisms in plants have been extensively reviewed in Madison et al. and Wang et al.^16,17^

### Diatoms sense rapid increases in P availability via Ca^2+^ signalling

While mechanisms for P limitation survival are relatively well studied, little is known about how P-starved diatoms sense and coordinate recovery following an influx of phosphate. However, a recent study by Helliwell et al.^19^ identified a role for Ca^2+^, the ubiquitous intracellular second messenger of eukaryotes, for sensing phosphate resupply (Figure 2).^19^ Employing a transgenic strain of *P. tricornutum* expressing the highly sensitive genetically encoded fluorescent Ca^2+^ biosensor (R-GECO1) this study revealed that when grown under P limitation, and subsequently resupplied with phosphate, *P. tricornutum* cells showed a rapid influx of Ca^2+^ into the cytosol. Inhibition of this response using the Ca^2+^ channel inhibitor Ruthenium Red impaired the ability of cells to coordinate key physiological adaptations driving recovery from P limitation, including rapid N uptake responses identified to follow phosphate resupply.

The diatom P-Ca^2+^ signaling response is elicited only in P-limited and not P replete cells, following phosphate resupply. In addition, other environmentally relevant organic forms of P besides phosphate induced the P-Ca^2+^ signal when resupplied, such as adenosine triphosphate (ATP) and D-glucose-6-phosphate (G6P). However, as the poorly hydrolysable form of ATP, adenosine 5’-(3-thiotriphosphate) (ATP-γ-S), did not cause [Ca^2+^]_cyt_ elevations this suggests that phosphate must be hydrolyzed from organic P forms to evoke the Ca^2+^ signaling response. It remains unknown how phosphate is sensed, i.e. by a phosphate sensor on the plasma membrane or an internal sensor (e.g. SPX) that detects changes in intracellular P stores.

## Diatoms do it differently?

Photosynthetic eukaryotes represent widely divergent taxa. Land plants (as well as green, red and glaucophyte algae) belong to the Archaeplastida, whereas diatoms are affiliated to an entirely different eukaryotic supergroup (the Stramenopile-Alveolate-Rhizaria or ‘SAR’ clade.^9^ Nevertheless, the importance of Ca^2+^-signaling for nutrient perception is steadily gaining recognition in divergent photosynthetic eukaryotes. Early reports identified sustained Ca^2+^ elevations (over minutes) in Fe deplete *P. tricornutum* cells following Fe resupply.^20^ Similarly, evidence for a role for Ca^2+^ signaling for sensing nitrate^21,22^ and K^+23^ has also recently emerged in vascular plants. However, despite exogenous ATP evoking cytosolic Ca^2+^ levels in plant and animal cells,^24–26^ this occurs independently of phosphate status, and so far, there is no evidence for a ‘diatom-like’ P-Ca^2+^ signaling response in P deplete plant cells. Similarly, the green alga *Chlamydomonas reinhardtii* does not appear to use Ca^2+^ signaling for phosphate (nor ammonium) resupply sensing.^27^ However, despite this evidence suggesting the use of Ca^2+^ for P sensing may be unique to diatoms, insights from other eukaryotes including taxa more closely related to diatoms (e.g. *Phytophthora* and *Ectocarpus*)^28^ will undoubtedly guide further characterization of the molecular players underlying diatom P-Ca^2+^ signaling and nutrient acquisition mechanisms more broadly. For instance, following nitrate-induced Ca^2+^ signals in *Arabidopsis thaliana*, Ca^2+^ binds to Ca^2+^ sensor proteins, Ca^2+^ protein kinases (CPK), which phosphorylate a NIN-LIKE PROTEIN transcription factor inducing downstream gene expression involved in nitrogen assimilation and signaling^21^. Functional characterization of Ca^2+^ sensors in response to phosphate in *P. tricornutum* remains unexplored. However, Ca^2+^/calmodulin-dependent protein kinase (CAMK), Ca^2+^-dependent protein kinases (CPKs), Ca^2+^-ATPase and Calmodulin (CAM1) become elevated during P limitation suggesting a potential role in the downstream response to P-Ca^2+^ signals.^19^ How these Ca^2+^ sensors function to coordinate diatom recovery following phosphate resupply should now be examined further.

## Crosstalk between P-signaling and N metabolism modulate balanced N and P acquisition

Whilst the relevance of P-Ca^2+^signaling beyond diatoms remains to be determined, what is emerging as a common feature between diverse eukaryotes is the sophisticated interplay between N and P signaling pathways to balance acquisition of these essential nutrients.^10^ During P limitation, costly N assimilation is unnecessary, but the P-Ca^2+^ signaling pathway is associated with rapid increases in uptake and assimilation of nitrate within just 4 h following P resupply, highlighting tight regulation between N and P networks.^19^ Similarly, nitrate-dependent control of P signaling machinery (namely regulation of PHR1) in plants limits cellular investment in P acquisition machinery when nitrate is limited.^29–31^ Trypsin, known typically for its role as a digestive enzyme in animals, has also recently been identified as a regulator of N and P acquisition in diatoms.^32,33^ In *P. tricornutum*, the gene *TRP2* is strongly upregulated during P limitation, and functions to repress N acquisition and promote P uptake. However, the mechanics mediating this regulatory response and whether it intersects with P-Ca^2+^-signaling remains to be elucidated. Further work exploring the extent and mechanisms underlying nutrient crosstalk will help shed light on the importance of such regulatory mechanisms in driving diatom success in the oceans, and how they cope with co-limitation by multiple nutrients simultaneously, as is frequently seen in large areas of the ocean.^34^ This is particularly important considering evidence of changing nutrient patterns in marine ecosystems, including expansion of nutrient-poor oligotrophic regions^35^ and shifts toward P limitation in certain regions where diatoms can thrive (e.g. the North Sea).^36^ Finally, studying the impacts of other global change stressors (such as elevated pCO_2_, rising temperatures and light levels), is also necessary for understanding the consequences on phytoplankton nutrient signaling and acquisition mechanisms in future ocean conditions.^37^ Together, this could help better predict impacts on phytoplankton productivity and community structure.
